# Transcriptome Analysis of Resistant Cotton Germplasm Responding to Reniform Nematodes

**DOI:** 10.3390/plants13070958

**Published:** 2024-03-26

**Authors:** Chunda Feng, Salliana R. Stetina, John E. Erpelding

**Affiliations:** USDA Agricultural Research Service, Crop Genetics Research Unit, Stoneville, MS 38776, USA

**Keywords:** Gossypium, *Rotylenchulus reniformis*, gene expression, resistance genes, cotton germplasm

## Abstract

Reniform nematode (*Rotylenchulus reniformis*) is an important microparasite for Upland cotton (*Gossypium hirsutum* L.) production. Growing resistant cultivars is the most economical management method, but only a few *G. barbadense* genotypes and some diploid Gossypium species confer high levels of resistance. This study conducted a transcriptome analysis of resistant genotypes to identify genes involved in host plant defense. Seedlings of *G. arboreum* accessions PI 529728 (A2-100) and PI 615699 (A2-190), and *G. barbadense* genotypes PI 608139 (GB 713) and PI 163608 (TX 110), were inoculated with the reniform nematode population MSRR04 and root samples were collected on the fifth (D5) and ninth (D9) day after inoculation. Differentially expressed genes (DEGs) were identified by comparing root transcriptomes from inoculated plants with those from non-inoculated plants. Accessions A2-100 and A2-190 showed 52 and 29 DEGs on D5, respectively, with 14 DEGs in common, and 18 DEGs for A2-100 and 11 DEGs for A2-190 on chromosome 5. On D9, four DEGs were found in A2-100 and two DEGs in A2-190. For GB 713, 52 and 43 DEGs were found, and for TX 110, 29 and 117 DEGs were observed on D5 and D9, respectively. Six DEGs were common at the two sampling times for these genotypes. Some DEGs were identified as Meloidogyne-induced cotton (*MIC*) 3 and 4, resistance gene analogs, or receptor-like proteins. Other DEGs have potential roles in plant defense, such as peroxidases, programmed cell death, pathogenesis related proteins, and systemic acquired resistance. Further research on these DEGs will aid in understanding the mechanisms of resistance to explore new applications for the development of resistant cultivars.

## 1. Introduction

Reniform nematode (*Rotylenchulus reniformis* Linford and Oliveira) is an obligate parasite that has a wide host range, which hinders management of this pest in several important crop species [1]. The nematode is a sedentary, semi-endoparasite, and has become one of the most important nematode species present in soils of the cotton-producing regions of the United States [1,2]. In the southeast states, such as Mississippi, Alabama, and Louisiana, reniform nematode has replaced *Meloidogyne incognita* (root-knot nematode) as the major nematode species infecting cotton [1,3]. Female vermiform nematodes can infect cotton roots throughout the growing season [4] and during this process, the nematode will modify host cells at the feeding site to form a syncytium, which provides the sole nutrient source for nematode development and reproduction [5]. Feeding on the root system interferes with the uptake of water and nutrients by the plant, resulting in stunting, delayed maturity, and yield reductions [4,6]. Moreover, root damage from nematode feeding makes cotton plants more vulnerable to soilborne diseases such as Fusarium wilt, which can compound yield losses [7]. In 2022, it was estimated that yield losses caused by reniform nematodes were 36.67 thousand metric tons, which was 1.2% of the U.S. total cotton production, and represents a loss of USD 74 M [8]. Yield losses in specific regions of the country were, however, significantly higher than the nationwide average with losses as high as 50% reported [9,10].

Limited approaches are available to manage reniform nematodes in Upland cotton (*Gossypium hirsutum* L.) production. Nematicide soil fumigation, seed treatment, in-furrow and foliar applications have been used to mitigate yield losses [1,10]. However, only a few nematicides are currently available and their efficacy can vary based on growing conditions [11,12,13,14]. Nematicides are effective in providing early season protection, but the nematode population can quickly and dramatically increase during the growing season due to the nematode’s short life cycle and high reproduction rate [1]. Additionally, nematicides are expensive and not all nematicide applications are profitable [14,15]. Human health and environmental concerns also need to be considered with the use of nematicides. Nematode management using crop rotation with non-host crops, such as corn and peanuts, or with resistant host cultivars, such as those available for soybean, has been recommended to reduce nematode populations [11,16]. This approach will require long rotational cycles to significantly lower the nematode population and therefore crop rotation is not always feasible due to the economic and resource constraints associated with cotton production [1,11]. Growing resistant Upland cotton cultivars or combining the application of nematicides with the use of resistant cultivars has been reported as an effective management strategy [1,11,17,18,19]. McCulloch et al. (2021) reported that resistant cultivars significantly suppress the reniform nematode population resulting in a 26% seedcotton yield increase compared to susceptible controls [20], whereas Koebernick et al. (2021) reported 8–20% yield increase using a resistant cultivar with a nematicide application during planting [17].

The development of resistant Upland cotton cultivars for reniform nematode management will require the use of multiple sources of resistance to limit the ability of the nematode to overcome a single source of resistance. Upland cotton accounts for 97% of cotton production in the U.S. (https://www.ers.usda.gov) (accessed on 1 October 2023); however, extensive screening of the Upland cotton germplasm collection only identified a few sources with moderate resistance [21,22,23,24]. These sources of resistance showed an inconsistent response [21,25] and have not been employed in commercial cultivar development. More desirable sources of resistance have been identified from tetraploid species *G. barbadense* L. [21,22] and several diploid Gossypium species, including *G. aridum* (Rose & Standl.) Skovst, *G. arboreum* L., *G. herbaceum* L., and *G. longicalyx* Hutch. & Lee [21,26,27,28].

Introgression of reniform nematode resistance from Gossypium germplasm into Upland cotton has been the focus for multiple breeding programs. Screening of *G. barbadense* germplasm collections identified a few accessions showing high levels of resistance [21,22] and multiple Upland cotton germplasm lines with improved resistance to reniform nematode have been released using two *G. barbadense* resistance sources. The *G. barbadense* accession TX 110 was used to develop two Upland cotton lines, TAM RKRNR-9 (Reg. No. GP-941, PI 662039) and TAM RKRNR-12 (Reg. No. GP-942, PI 662040), which showed suppression of reniform nematode reproduction by 40–70% [29]. The most widely used source of resistance has been the *G. barbadense* accession GB 713 and breeding lines derived from this source of resistance have been used in commercial cultivar development. Three lines, namely M713Ren1 (Reg. No. GP-958, PI 665928), M713Ren2 (Reg. No. GP-959, PI 665929), and M713Ren5 (Reg. No. GP-960, PI 665930), were developed in Mississippi and showed approximately 90% suppression in reniform nematode egg production [30]. The lines BARBREN-713 (Reg. No. GP-987, PI 671965) and BARBREN-713-32 (Reg. no. GP-1134, PI 701076) [31,32] were developed in Texas and suppressed nematode egg production by 74–92% [32]. Three QTLs controlling reniform nematode resistance were mapped for GB 713 with QTLs *Ren^barb1^* and *Ren^barb2^* located on chromosome AD_ch21_Dt.11 and QTL *Ren^barb3^* on chromosome AD_ch18_Dt.13 [33]. A recent study has shown that two QTLs controlled resistance with QTL *Ren^barb2^* mapped to a 17.7 MB interval (36.5–54.2 MB) on AD_ch21_Dt.11 and QTL *Ren^barb3^* mapped to a 1.8 MB interval (5.9–7.7 MB) on AD_ch18_Dt.13 in the genome assembly of BARBREN-713-32 [34]. Resistance was associated with QTL *Ren^barb2^*, whereas QTL *Ren^barb3^* did not provide resistance, but was required to recover the high level of resistance characteristic of GB 713 [35,36].

The diploid cotton species are also a potential source of novel resistance genes; however, transferring resistance to Upland cotton is technically demanding and laborious. *G. longicalyx* was the only cotton species reported to show an immune response to reniform nematode infection [21]. This source of resistance was successfully transferred to Upland cotton using the bridging lines (*G. hirsutum* × *G. longicalyx*) and (*G. hirsutum* × *G. herbaceum*) with the breeding lines LONREN-1 (Reg. No. GP-977, PI 669509) and LONREN-2 (Reg. No. GP978, PI 669510) being developed [37,38,39]. The *G. longicalyx* resistance gene *Ren^lon^* was mapped on chromosome AD_ch21_Dt.11 [40]. Resistance derived from *G. longicalyx* was associated with a hypersensitive response resulting in root necrosis and severe plant stunting, therefore this resistance source has not been used for commercial cultivar development [41,42]. Bhandari et al. (2015) [43] reported hypersensitive reactions in *G. hirsutum* germplasm lines LONREN-1 and LONREN-2 with *G. longicalyx* resistance, and *G. arboreum* accession A2-190, to two isolates from Louisiana based on plant height reductions; however, reductions in root growth for A2-190 was not reported. Severe root necrosis associated with resistance has not been reported for other diploid cotton species showing high levels of resistance, although, limited research has been conducted on these resistant sources. Resistance from *G. aridum* and *G. arboreum* were transferred to Upland cotton using the (*G. hirsutum* × *G. aridum*) bridging line [27,44]. The resistance QTL *Ren^ari^* derived from *G. aridum* was mapped to AD_ch21_Dt.11 in Upland cotton [27] and at least two resistance genes were reported for the *G. arboreum* source of resistance [44]. Limited breeding research has been conducted on these resistance sources and no Upland cotton breeding lines have been released.

The *G. arboreum* germplasm collection includes more than 1600 accessions and resistant genotypes have been frequently observed in this collection [28]. A few *G. arboreum* accessions have been genetically characterized for nematode resistance with resistance being conferred by one or a few genes [45,46,47]. A genome-wide association study of 246 accessions revealed 15 SNPs significantly associated with reniform nematode resistance [48]. The lack of genomic tools has hindered the transfer of resistance from *G. arboreum* to Upland cotton. To enhance the utilization of resistance sources, in the present study, the transcriptomes of *G. arboreum* and *G. barbadense* resistant genotypes responding to reniform nematode were evaluated to identify differentially expressed genes and genomic regions potentially associated with nematode resistance.

## 2. Results

### 2.1. Sequencing Results

RNA sequences were obtained for 47 of the 48 treatments due to library construction failing for 1 sample of the non-inoculated GB 713 for the D9 treatment. The number of raw reads for each sample ranged from 19.64 to 38.04 M for single-end sequencing and 61.08 to 74.75 M for paired-end sequencing, with an average of 28.61 M raw reads. The number of bases generated from each sample ranged from 1.98 to 3.84 giga bases (Gb) for single-end sequencing and 6.17 to 7.55 Gb for paired-end sequencing, with an average of 2.89 Gb. Over 96% of bases had quality scores Q20 or higher (error *p* ≤ 0.01) and 93% of bases had quality scores Q30 or higher (error *p* ≤ 0.001). The average undetermined bases (N) were 0.03%.

### 2.2. Differential Expression of Genes in A2-100

For the D5 treatment, 29,168 genes were expressed with 57 genes differentially expressed between the inoculated and non-inoculated treatments. Expression was upregulated for 56 of these genes. The expression levels for 52 genes changed by at least two-fold. At least 1 differentially expressed gene (DEG) was observed on each of the 13 chromosomes, with 18 DEGs recorded for chromosome A05 (Table 1). Five DEGs located on chromosome A05 and two on chromosome A12 had uncharacterized functions. Disease resistance related genes were observed, including 11 genes encoding receptor-like proteins and 2 genes encoding Meloidogyne-induced cotton (*MIC*) 3 and 4 proteins (Table 2). Genes that play important roles in plant immune and defense reactions were also recorded, such as plant programmed cell death (one metacaspase-9-like, two ervatamin-B-like, and one self-pruning), disease resistance reactions (three peroxidase, two hevamine-A-like genes, one glucan endo-1,3-beta-glucosidase 7-like), and systemic acquired resistance (one protein NIM1-interacting 1). In addition, four transcript factors were differentially expressed.

Data from the D9 treatment showed 4 out of the 29,072 genes were differentially expressed. The expression levels were 4 to 32 times higher in the inoculated treatment compared to the non-inoculated control and these DEGs were upregulated. Two of the DEGs were associated with chromosome A05 (Table 1) and they also showed differential expression for the D5 treatment. These four DEGs showed functions associated with plant defense (Table 2).

### 2.3. Differential Expression of Genes in A2-190

A total of 29,239 genes were expressed for the D5 treatment with 56 genes differentially expressed between the inoculated and non-inoculated treatments, and 44 of these DEGs were upregulated. The expression levels for 29 DEGs showed more than a two-fold change with 25 DEGs showing upregulation. The 29 DEGs were distributed on 9 chromosomes, including 11 genes located on chromosome A05 (Table 1). Two DEGs associated with resistance to nematodes, Meloidogyne-induced cotton genes (*MIC-3* and *MIC-4*), were observed (Table 2). Several other DEGs have functions associated with plant defense and disease resistance, including two resistance gene analogs (*DMR6*-like), three receptor-like kinase genes, peroxidase-like, hevamine-A-like, jasmonate-induced protein, and transcription factor *MYC2* (*XM_017774272.2*). Six DEGs on chromosome A05 and two on chromosome A10 were uncharacterized for gene function.

From the 29,096 genes expressed for the D9 treatment, 2 genes, residing on chromosomes A05 and A12, were differentially expressed (Table 1). Expression levels increased 5 and 11 times in the inoculated treatment compared to the non-inoculated control. These DEGs had functions associated with plant defense (Table 2).

### 2.4. Differential Gene Expression for TX 110

For the D5 treatment, expression was detected for 65,249 genes. A total of 31 of these genes were differentially expressed between the inoculated and non-inoculated treatments with 27 DEGs upregulated. The change in expression ranged from 2- to 120-fold for 29 DEGs and these genes were distributed on 18 chromosomes (Table 3). DEGs were identified on chromosomes of the A and D subgenomes with a single DEG per chromosome more frequently recorded. Five of these DEGs showed gene functions associated with plant defense (Table 4) and three genes were transcription factors. No gene function was recorded for the majority of the DEGs.

A total of 64,499 genes showed expression for the D9 treatment with 119 genes differentially expressed between inoculated and non-inoculated treatments, including 82 genes upregulated. At least a two-fold change in expression was recorded for 117 DEGs. The 117 genes were distributed on all 26 chromosomes (Table 3). Multiple DEGs were detected on each chromosome with nine genes observed on chromosomes AD_ch09_At.09 and AD_ch26_Dt.12. Twenty-nine DEGs had functions associated with plant defense (Table 4). Additionally, several transcription factors were identified. However, the gene function for the majority of the DEGs was uncharacterized.

### 2.5. Differential Gene Expression for GB 713

Gene expression analysis identified 65,316 genes for the D5 treatment using the genome of Pima 90 as reference. Overall, 66 genes were differentially expressed with 61 DEGs upregulated. Fifty-two of these DEGs showed at least a two-fold change in expression levels between inoculation treatments. The 52 DEGs were distributed across the 26 chromosomes with a relatively small number located on each chromosome (Table 3). The function for the majority of the DEGs was uncharacterized. Four DEGs showed a function associated with plant defense (Table 5) and transcription factors, such as *Myb*, were also observed. For the D9 treatment, 43 genes were differentially expressed from a total of 64,073 genes showing expression. Expression levels of these DEGs varied from 2- to 48-fold with 28 genes upregulated. DEGs were distributed on 19 chromosomes, including 6 genes on AD_ch05_At.05 and 5 on chromosome AD_ch11_At.11 (Table 3). Eight DEGs showed functions association with plant defense, including one resistance gene analog (Table 5). Two transcription factors were also differentially expressed, but most DEGs were uncharacterized.

From the 58,851 genes showing expression for the D5 treatment using BARBREN713 genome as reference, 61 were differentially expressed with 55 upregulated. A total of 50 genes showed a 2- to 18-fold change in expression levels. These 50 DEGs were associated with 22 chromosomes, including 6 genes on chromosome AD_ch21_Dt.11 (Table 3). Three of the DEGs showed functions associated with plant defense (Table 5). A total of 2 transcription factors were identified and 17 DEGs had an undetermined function. For the D9 treatment, 39 genes were differentially expressed from the 57,510 genes detected with 25 genes upregulated. Expression levels varied from 2- to 40-fold. DEGs were associated with 21 chromosomes, including 5 genes on chromosome AD_ch26_Dt.12 and 3 each on chromosomes AD_ch05_At.05, AD_ch08_At.08, AD_ch10_At.10, AD_ch24_Dt.08, and AD_ch21_Dt.11 (Table 3). Seven DEGs showed functions associated with plant defense, including resistance gene analogs and a Meloidogyne-induced cotton gene (*MIC-3*) (Table 5).

Of the 60,529 genes observed for the D5 treatment using BARBREN713-32 genome as reference, 41 genes were differentially expressed with 38 genes upregulated. These genes were distributed on 19 chromosomes, including 5 on chromosome AD_ch07_At.07 and 4 on chromosome AD_ch05_At.05 (Table 3). The functions of 17 genes remained unknown. One transcription factor and two genes involved in abiotic responses were identified; however, no DEGs associated with plant defense were identified. For the D9 treatment, 25 genes showed differential expression from a total of 44,266 genes evaluated with expression for 16 DEGs upregulated. These 25 DEGs were associated with 15 chromosomes with three located on chromosome AD_ch05_At.05 (Table 3). Three DEGs showed functions associated with plant defense (Table 5).

### 2.6. Comparisons of Differential Expression within and between Gossypium Species

A total of 14 genes were differentially expressed for the D5 treatment in A2-100 and A2-190 with 7 DEGs mapped to chromosome A05 (Table 6). Six DEGs showed functions associated with disease resistance, including Meloidogyne-induced cotton genes *MIC-3* (*XM_017750577*) and *MIC-4* (*XM_017750341*), peroxidase (*XM_017747283*), hevamine-A-like (*XM_017781096*), metacaspase-9-like (*XM_053030089*), and early-nodulin-75-like (*XM_17753691*) genes with three DEGs mapped on chromosomes A05. One gene (*XM_017773121.2*) showed differential expression for the D5 and D9 treatments in A2-100 and for the D9 treatment in A2-190 and was mapped on chromosome A05. This aspartyl protease gene has an important role in plant defense.

Twelve genes were differentially expressed in TX 110 and GB 713 using Pima 90 as a reference genome (Table 7). Three genes (*GbM_A02G0072*, *GbM_A01G0832*, and *GbM_D01G0797*) showed differential expression across the genotypes on D5 with two genes (*GbM_A04G0525* and *GbM_A13G1710*) showing differential expression on D9. On D5 and D9, three genes showed differential expression for TX 110, but only showed differential expression on one of the sampling treatments for GB 713. Two genes (*GbM_A11G3523* and *GbM_A10G2857*) were differentially expressed across the genotypes on D5 and D9.

Using the functional annotation of the DEGs as a comparison across species and genotypes, some similarity in disease resistance related genes were observed. For example, receptor-like proteins, peroxidase, hevamine-A-like, and glucan endo-1,3-beta-glucosidase were differentially expressed across the four genotypes. Metacaspase-9-like and aspartyl protease were differentially expressed for *G. barbardense* TX 110 and for *G. arboreum* A2-100 and A2-190, whereas the Meloidogyne-induced cotton gene *MIC-3* was differentially expressed for *G. barbardense* GB 713 and for A2-100 and A2-190.

## 3. Discussion

The evolutionary and commercial breeding history for Upland cotton has resulted in a narrow germplasm pool, which makes current cultivars vulnerable to changes in pest populations. Although reniform nematode was first identified in cotton in 1940, release of resistant commercial cultivars has only occurred within the past five years. The development of resistant cultivars has been hindered by the lack of resistance genes within Upland cotton germplasm, the difficulty in screening for reniform nematode resistance, and the complex inheritance of resistance. Related cotton species are a valuable resource of resistance genes; however, genetic and genomic evaluations are essential to identify genes associated with resistance to develop marker-assisted selection strategies.

Gene expression studies are one approach to identify genes associated with resistance. Transcriptome analysis of *G. hirsutum* cultivars that are susceptible (DP90 and SG747), resistant (BARBREN-713) and hypersensitive (LONREN-1) to reniform nematodes revealed many DEGs associated with resistance, such as cell wall architecture, hormone metabolism and signaling, ROS levels, cell death pathways, and pathogenesis [49]. In the present study, DEGs with a range of functions were identified from the four cotton genotypes in response to reniform nematode infection. Resistant gene analogs and non-specific resistance genes, such as chitinase, glucan endo-1,3-beta-glucosidase, and pathogenesis-related proteins, were differentially expressed and may have an important role in response to reniform nematode infection. The DEGs were more often upregulated than downregulated, higher expression of certain genes may help to fight the parasites and mitigate the damage of nematodes. Li et al. (2015) [49] reported that more DEGs were downregulated for GB 713, whereas the other two evaluated genotypes show slightly more DEGs being upregulated.

Meloidogyne induced cotton genes *MIC-3* and *MIC-4* were differentially expressed in A2-100 and A2-190, and MIC-3 gene was differentially expressed for GB 713, and they were upregulated in these genotypes. *MIC-3* was found to be specifically expressed in cotton resistant genotypes when inoculated with *M. incognita* [50]. Overexpression of *MIC-3* in *G. hirsutum* cv. Coker 312 reduced egg production of *M. incognita* by 60–75%, but no effect on reniform nematode reproduction was reported [51]. These genes showed expression in A2-100 and A2-190 on D5, which may indicate that expression of these genes occurs early in the infection process and may influence the establishment of the feeding site. In contrast, *MIC-3* expression was observed in GB 713 on D9, suggesting a possible role in hindering nematode development. These DEGs were associated with chromosome A05 in both species. Several cotton resistance genes/QTLs to biotic and abiotic stresses have been mapped on chromosome AD-ch05_At.05 [52,53,54], which may result from natural selections in cotton evolution. The physical locations on cotton genomes for these genes/QTLs were unknown, thus their distance to the resistance genes identified in this study are undetermined.

Genes for receptor-like proteins showed expression for A2-100 and A2-190 on D5 and in GB 713 on D9. These DEGs were upregulated for these genotypes. Receptor-like proteins are cell surface receptors composed of several distinct domains, including signal peptide, extracellular leucine-rich repeat (LRR) region, and transmembrane domain, with these proteins playing an important role in plant development as well as disease resistance [55]. DEGs of receptor-like proteins were associated with chromosome A01 across the cotton genotypes in the present study, which may suggest a conservation response. Additionally, Li et al. (2018) [48] reported one SNP significantly associated with reniform nematode resistance for chromosome A01 in *G. arboreum*, which is about 9 Mb from the receptor-like gene *XM_053030958.1* (Table 2).

Hevamine-A-like protein is a type of plant chitinase and lysozyme that are important for plant defense against pathogens [56]. Chitin is an essential component of the nematode eggshell and pharynx, and the disturbance of chitin synthesis or hydrolysis could lead to nematode embryonic lethality, defective egg laying, or molting failure [57]. Upregulated differential expression for hevamine-A-like protein was recorded for the D5 treatment across the four genotypes and for the D9 treatment in A2-190 and TX 110. This DEG was commonly associated with chromosome A12 across the four genotypes. A genome-wide association study of *G. arboreum* genotypes also identified candidate genes for reniform nematode resistance on chromosome A12 [48].

Several other genes associated with plant defense were also recorded for the genotypes evaluated in the current study. Multiple peroxidase genes were differentially expressed for A2-100, A2-190, and GB 713, which were upregulated. Peroxidase genes are critical for cell wall stiffening [58]. Li et al. (2015) [49] reported peroxidase genes were differentially expressed in response to reniform nematode infection across the four genotypes that showed different infection responses. Aspartyl protease family protein At5g10770-like showed differential expression and was upregulated for A2-100, A2-190, and TX 110. This protein could degrade pathogen effectors contributing to reducing virulence and degrade pathogenesis-related proteins that would induce the expression of genes involved in stress and defense responses, innate immunity, and systemic acquired resistance (SAR) [59]. A metacaspase-9-like gene was recorded for A2-100 and TX 110, and two ervatamin-B-like genes and one self-pruning gene were observed for A2-100. These genes have critical roles in programmed cell death during plant development and defense responses [60,61,62]. Two genes differentially expressed for A2-100 and one gene for TX 110 encode early-nodulin like proteins. These proteins were reported to have an important role for enhancing fitness of the pathogen during host colonization [63]. In addition, these proteins have functions associated with the transportation of nutrients and plant development [63]. One DEG (*XM_017774501.2*) recorded for A2-100 encodes the NIM1-interacting protein. This gene has an important role in systemic acquired resistance [64]. Two Kunitz trypsin inhibitor 5 genes were differentially expressed for TX 110. These genes were reported to have functions associated with the protection of plants from insect predators [65]. One nematocidal crystal cry1Ag gene observed for TX 110 may have resulted from *Bacillus thuringiensis* contamination [66].

Mapping reniform nematode resistance genes will be critical for the development of molecular markers to enhance the introgression of these genes from related Gossypium species for the development of resistant Upland cotton cultivars. Fifteen SNPs significantly associated with reniform nematode resistance were mapped to eight chromosomes for *G. arboreum* [48]. DEGs reported in the current study were widely distributed across the genome with DEGs observed on 13 chromosomes for A2-100 and 9 chromosomes for A2-190; however, the majority of the DEGs were associated with chromosome A05 for the 2 genotypes. Thus, DNA markers developed from chromosome A05 should be selected for screening segregating populations for nematode resistance derived from *G. arboreum* sources. DEGs associated with chromosome A12 may also have a role in nematode resistance for the *G. arboreum* genotypes. Some similarities were observed for the DEGs across the genotypes and these data may suggest some shared resistance genes; however, identifying *G. arboreum* genotypes with unique resistance genes will be necessary to increase genetic diversity.

DEGs were also observed on chromosome A05 of the A-genome and the homoeologous chromosome 19 of the D-genome for TX 110 and GB 713 with more DEGs frequently associated with these chromosomes for the D9 treatment. DEGs were distributed across most of the chromosomes for these two genotypes, but the frequency of DEGs associated with individual chromosomes varied across genotypes and across inoculation treatments. The occurrence of DEGs across the genome may suggest expression of genes related to root tissue damage and not associated with nematode resistance. The resistance QTLs transferred from GB 713 were mapped to AD_ch21_Dt.11 and AD_ch18_Dt.13 in the genome assembly of BARBREN-713-32 [34]. Transcriptome analysis of GB 713 and TX 110 in the current research identified DEGs associated with these two chromosomes and the DEGs were in or adjacent to the intervals identified for BARBREN-713-32. Two DEGs for TX 110 and one for GB 713 that were mapped onto these chromosomes had functions associated with plant defense. A few DEGs were comparable across the two genotypes and may indicated some similarity in the mechanism of resistance.

Sampling time is very critical for the transcriptome analysis of cotton responding to reniform nematodes. While inspecting nematode infection on a small subset of plants two DAI, it was found that not all plants were infected, and the *G. arboreum* accessions had little root development. All sampled plants were infected on D5, and plant root mass was enough for RNA extraction. The parasitizing nematodes were in the gravid stage on D9. Therefore, root samples were collected on D5 and D9 in this study. A comparison across the two inoculation treatments showed different gene expression patterns among the genotypes evaluated in the present study. The number of DEGs recorded for the D9 treatment was dramatically reduced compared to the D5 treatment for the *G. arboreum* genotypes. These data could suggest resistance is associated with the early stages of nematode infection. However, fewer DEGs were observed for A2-190 for the two inoculation treatments compared to A2-100. This may suggest that resistance associated with A2-190 could hinder the establishment of the feeding site for the nematode, resulting in the low number of nematodes typically observed on the root system for this genotype. Collecting root samples prior to D5 may aid in the identification of additional DEGs associated with resistance. However, transcript evaluations prior to D5 may require a different sampling approach because of the few nematodes infecting the root system for highly resistant genotypes and low root weights characteristic of *G. arboreum* accessions. In contrast, resistance associated with A2-100 may occur later in the infection process, leading to a greater number of nematodes infecting the root system [67]; thus, contributing to the moderately resistant response recorded for this genotype and the higher number of DEGs observed. The difference in the number of DEGs across inoculation treatments was reduced for the *G. barbadense* genotype GB 713; although, fewer DEGs were recorded for the D9 treatment. These data could suggest a different mode of resistance compared to the *G. arboreum* genotypes. This trend was reversed for TX 110 with more DEGs recorded for the D9 treatment than the D5 treatment. A moderately resistant response to reniform nematode infection has been reported for TX 110 [22]. When infected with the MSRR04 reniform nematode population, TX 110 and GB 713 showed similar nematode development for two days after inoculation; then, more rapid progression of nematode development was observed for TX 110 compared to GB 713 [67]. The expression of many non-specific pathogenesis-related genes was also observed for TX 110 for the D9 treatment in the current study, which could be associated with the moderately resistant response. The availability of data on nematode development for the two genotypes could be used in future studies to select additional time points to evaluate changes in gene expression to assess the pathways contributing to the resistant response.

Reference genomes are critical for transcriptome analysis. ShiXiYa 1 (PI 615743) was reported to have a moderately resistance response to reniform nematode infection [28]. Using this reference genome, many receptor-like DEGs were identified from the two *G. arboreum* genotypes evaluated in the current study. While using the Pima 90 genome as a reference, relatively less receptor-like DEGs were identified from the two *G. barbadense* genotypes; this could result from the lack of resistance in Pima 90 to reniform nematode (its response to reniform nematode needs to be tested). Three reference genomes were used for gene identification and feature count when analyzing the transcriptomes for GB 713. The number of non-specific plant defense related DEGs recorded was reduced using the *G. hirsutum* genotypes BARBREN 713 and BARBREN 713-32 resistant to reniform nematodes. Since their resistance was derived from GB 713, it is possible that these DEGs of GB 713 confer resistance to reniform nematode. Three DEGs were found in each of the *G. hirsutum* reference genomes, suggesting some resistance genes are common in these lines. The discrepancy in the numbers of DEGs identified may result from the selections in breeding or the difference of genome assemblies and annotations.

DEGs associated with disease resistance genes identified from the current research will be further tested using other resistant and susceptible cotton genotypes to determine their role in suppressing reniform nematode infection and development. More than 100 resistant genotypes were identified from the *G. arboreum* germplasm collection [28] and would be useful for these evaluations. Accessions from this collection showed a wide range of variations for infection response, which could be useful for identifying additional DEGs associated with nematode resistance. Some accessions showed very low numbers of nematodes infecting the root system and resistance could be associated with the establishment of the feeding site, whereas hindering nematode egg production could be associated with host resistance for other accessions. Increasing root sampling times would also be useful to identify additional DEGs associated with this variation in infection response although constitutively expressed resistance genes cannot be identified using transcriptome analysis. Molecular markers for resistance genes can be developed using segregating populations, which would be beneficial for the introgression of resistance in Upland cotton breeding programs.

## 4. Materials and Methods

### 4.1. Plant Materials, Reniform Nematode Inoculation, and RNA Sequencing

Two *G. arboreum* accessions A2-100 (PI 529728) and A2-190 (PI 615699), and two *G. barbadense* accessions TX 110 (PI 163608) and GB 713 (PI 608139) were used for this study. These accessions were originally obtained from USDA-ARS Germplasm Resource Information Network (GRIN) and have been self-pollinated over multiple generations at Stoneville, MS or at the Cotton Winter Nursery, Tecoman, Mexico to develop homogenous breeding lines. The reniform nematode response for these genotypes has been evaluated across multiple experiments. The A2-100 genotype showed a moderately resistant reaction [45], TX 110 showed moderate to high resistance when tested with different reniform nematode populations [21,22,68], whereas genotypes A2-190 and GB 713 showed a highly resistant response [22,44]. A susceptible *G. hirsutum* cultivar Deltapine 16 (PI 529251) was also included solely for checking inoculation.

These genotypes were inoculated with the Mississippi reniform nematode population MSRR04 [69] following the procedure described by Stetina et al. (2014) [70]. Briefly, a single seed was planted in a conical plastic pot (Ray Leach SL-10 Cone-tainer, Stuewe & Sons, Inc., Tangent, OR, USA) containing 120 cm^3^ of a steam-sterilized soil mixture of one-part sandy loam soil and two-parts sand. For this study, 60 seeds were planted for each genotype and the experiment was conducted in 2 growth chambers. One chamber was used for the inoculation treatment, whereas the second chamber was used for the non-inoculated control treatment. Growth conditions for the experiment were set to a constant temperature of 28 °C with a 16 h photoperiod. Genotypes were arranged in a completely randomized design with 3 replications and 10 pots per replication. Seven days after planting, the nematode inoculation treatments were conducted by infesting the soil in each pot with approximately 1000 nematodes (mixed vermiform life stages). On the fifth (D5) and ninth (D9) day after inoculation (DAI), three plants of each replication for each genotype were randomly selected to harvest root samples. The corresponding control samples were also harvested at each time interval. Briefly, the seedlings were removed from the pots and the soil was washed from the roots. The shoot was removed from the root system below the soil line and the roots were further washed with sterilized reverse osmosis water. The root systems from the three selected plants were combined and ground to a fine powder in liquid nitrogen and then transferred to 25 mL tube for storage at −70 °C. RNA extraction was conducted using the Spectrum Plant Total RNA kit (Sigma-Aldrich Inc., St. Louis, MO, USA) and quantified using a spectrophotometer. Libraries were constructed from total RNA using the Illumina TruSeq RNA Sample Preparation Kit v2 (Illumina, Inc., San Diego, CA, USA) with set B indexed adapter sequences. The transcriptomes were sequenced with Illumina Hiseq 1000 platform at MacroGene, Seoul, Korea. Two flowcells were used for sequencing with 42 single-end sequenced samples and 6 paired-end sequenced samples.

### 4.2. Data Analysis

Data analysis was conducted on the SCINet high performance computing platform (scinet.usda.gov). The raw RNA reads were checked with BBduk (sourceforge.net/projects/bbmap/, version 39.03) to remove adapters and low-quality bases (*p* = 0.1), then reads were aligned to reference genomes using the software Spliced Transcripts Alignment to a Reference (STAR, version 2.7.11b) [71] to identify expressed genes and count the features following the software manual. The A2 (*G. arboreum*) reference genome of ShiXiYa 1 (PI 615743) was used to analyze the transcriptomes for A2-100 and A2-190 [72]. The reference genome for Pima90 (*G. barbadense*) was used to analyze the transcriptomes for TX 110 and GB 713 [73]. In addition, the reference genomes of *G. hirsutum* germplasm lines BARBREN-713 (B713) and BARBREN-713-32 (Bar32), whose reniform nematode resistance was derived from GB 713, were used to analyze the transcriptomes of GB 713 [34]. The genomic sequences and transcriptomes of these reference genomes were downloaded from CottonGen (https://www.cottongen.org) (accessed on 15 June 2023) [74]. The transcripts aligned to the reference genomes and their feature counts derived from STAR analysis were further used to identify DEGs with the R package DESeq2 (adjusted *p* < 0.05) [75]. DEGs with at least a two-fold change of expression levels were further analyzed. The sequences and chromosomal locations of the DEGs were obtained from the relevant transcript sequences of the reference genomes and the potential functions of the DEGs were determine by searching against the nr database (https://www.ncbi.nlm.nih.gov) (accessed on 31 August 2023) with BLASTX (*p* < 10^−6^).

## 5. Conclusions

In this study, the transcriptome analysis of four resistant Gossypium genotypes challenged with reniform nematodes revealed differentially expressed genes and their chromosomal locations. Plant defense related genes, including nematode resistant genes, were identified. Some plant defense genes were common within and between Gossypium species. Further study of the identified resistance genes may help to understand the mechanism of resistance and to develop resistant cotton cultivars.

## Figures and Tables

**Table 1:** Number of DEGs and their chromosome locations for *G. arboreum* genotypes A2-100 and A2-190 evaluated on the fifth (D5) and ninth (D9) day after inoculation with reniform nematodes.

Chromosome	A2-100		A2-190	
	D5	D9	D5	D9
A01	3	0	1	0
A02	4	0	2	0
A03	1	1	0	0
A04	1	0	0	0
A05	18	2	11	1
A06	4	0	2	0
A07	5	0	0	0
A08	2	0	2	0
A09	1	1	0	0
A10	3	0	5	0
A11	2	0	2	0
A12	7	0	2	1
A13	1	0	2	0
total	52	4	29	2

**Table 2:** Potential plant defense related DEGs and associated chromosomal location for *G. arboreum* genotypes A2-100 and A2-190 evaluated on the fifth and ninth day after inoculation (DAI) with reniform nematodes.

Line	DAI	Chromosome	Gene Name	Function
A2-100	5	A01	*XM_017784407.2*	receptor kinase-like protein *Xa21*
A2-100	5	A01	*XM_053030958.1*	receptor-like protein 6 isoform *X4*
A2-100	5	A03	*XM_053026038.1*	receptor-like protein 14
A2-100	5	A04	*XM_017758709.2*	peroxidase *P7*-like
A2-100	5	A05	*XM_017747283.2*	peroxidase *P7*-like isoform X1
A2-100	5	A05	*XM_017750577.2*	Meloidogyne-induced cotton 3
A2-100	5	A05	*XM_017750341.2*	Meloidogyne-induced cotton 4
A2-100	5	A05	*XM_017784652.2*	ervatamin-B-like
A2-100	5	A05	*XM_017773121.2*	aspartyl protease family protein
A2-100	5	A05	*XM_017792281.2*	receptor-like protein 53
A2-100	5	A05	*XM_053027636.1*	receptor-like protein *EIX2*
A2-100	5	A05	*XM_017749381.1*	receptor-like protein *EIX2*
A2-100	5	A05	*XM_017792234.2*	LRR receptor-like *At3g47570*
A2-100	5	A05	*XM_053027631.1*	receptor-like protein *EIX2*
A2-100	5	A05	*XM_017750490.2*	cysteine-rich receptor-like protein kinase 2
A2-100	5	A05	*XM_017788880.2*	receptor-like protein kinase 5
A2-100	5	A06	*XM_053030089.1*	metacaspase-9-like
A2-100	5	A06	*XM_017785873.2*	early nodulin-like protein 2
A2-100	5	A06	*XM_053029232.1*	receptor-like protein 15 isoform *X3*
A2-100	5	A08	*XM_017763075.2*	cationic peroxidase 1-like
A2-100	5	A09	*XM_017754591.2*	glucan endo-1,3-beta-glucosidase 7-like
A2-100	5	A10	*XM_017753691.1*	early nodulin-75-like
A2-100	5	A11	*XM_017774501.2*	protein NIM1-interacting 1
A2-100	5	A11	*XM_017774657.2*	ervatamin-B-like
A2-100	5	A12	*XM_017781096.2*	hevamine-A-like
A2-100	5	A12	*XM_017780751.2*	hevamine-A-like
A2-100	9	A03	*XM_017777226.2*	annexin *Gh1*-like
A2-100	9	A05	*XM_017784652.2*	ervatamin-B-like
A2-100	9	A05	*XM_017773121.2*	aspartyl protease family protein
A2-100	9	A09	*XM_017754924.2*	protein self-pruning
A2-190	5	A01	*XM_017784262.1*	receptor-like protein 6
A2-190	5	A05	*XM_017765593.2*	protein *DMR6*-like oxygenase 2-like
A2-190	5	A05	*XM_017747283.2*	peroxidase *P7*-like isoform X1
A2-100	5	A05	*XM_017750577.2*	Meloidogyne-induced cotton 3
A2-190	5	A05	*XM_017750341.2*	Meloidogyne-induced cotton 4
A2-190	5	A05	*XM_017769610.2*	MDIS1-interacting receptor like kinase
A2-190	5	A06	*XM_053030089.1*	peroxidase *P7*-like
A2-190	5	A08	*XM_017768785.2*	Downy mildew resistance 6-like
A2-190	5	A10	*XM_017747283.2*	peroxidase *P7*-like isoform X1
A2-190	5	A10	*XM_053020047.1*	wall-associated receptor kinase-like 1
A2-190	5	A12	*XM_017781096.2*	hevamine-A-like
A2-190	9	A05	*XM_017773121.2*	aspartyl protease family protein
A2-190	9	A012	*XM_017781096.2*	hevamine-A-like

**Table 3:** Number of DEGs and their chromosome locations for *G. barbadense* genotypes TX 110 and GB 713 evaluated on the fifth (D5) and ninth (D9) day after inoculation with reniform nematodes using Pima 90, BARBREN 713 (B713), and BARBREN 713-32 (Bar32) reference genomes.

Chromosome	TX 110		GB 713					
	Pima 90		Pima 90		B713		Bar32	
	D5	D9	D5	D9	D5	D9	D5	D9
AD_ch01_At.01	3	4	1	2	1	1	1	1
AD_ch02_At.02	2	4	1	2	1	1	1	1
AD_ch03_At.03	1	2	2	0	2	1	4	1
AD_ch04_At.04	0	3	1	1	0	1	1	3
AD_ch05_At.05	0	5	3	6	3	3	4	3
AD_ch06_At.06	0	3	2	0	0	0	1	0
AD_ch07_At.07	0	2	3	1	2	1	5	1
AD_ch08.At.08	0	3	1	1	0	3	1	1
AD_ch09_At.09	1	9	1	2	1	1	1	1
AD_ch10_At.10	2	4	1	2	3	3	3	3
AD_ch11_At.11	1	8	3	5	2	0	2	1
AD_ch12_At.12	2	4	3	1	3	1	2	2
AD_ch13_At.13	1	6	1	3	1	2	1	3
AD_ch15_Dt.01	1	3	3	1	3	2	3	1
AD_ch14_Dt.02	1	2	2	0	3	0	3	0
AD_ch17_Dt.03	0	2	2	1	2	1	2	1
AD_ch22_Dt.04	3	4	2	2	2	2	2	2
AD_ch19_Dt.05	2	5	1	0	0	1	0	0
AD_ch25_Dt.06	1	5	4	0	3	0	2	0
AD_ch16_Dt.07	0	7	3	0	4	0	2	0
AD_ch24_Dt.08	1	4	3	1	1	3	0	0
AD_ch23_Dt.09	2	5	1	2	2	2	0	0
AD_ch20_Dt.10	1	6	1	0	1	1	0	0
AD_ch21_Dt.11	0	5	4	4	6	3	0	0
AD_ch26_Dt.12	2	9	1	4	2	5	0	0
AD_ch18_Dt.13	2	3	2	2	2	1	0	0
total	29	117	52	43	50	39	41	25

**Table 4:** Potential plant defense related DEGs and associated chromosomal location for the *G. barbadense* genotype TX 110 assessed on the fifth and ninth day after inoculation (DAI) with reniform nematodes.

DAI	Chromosome	Gene Name	Function
5	AD_ch09_At.09	*GbM_A09G1049*	glucan endo-1,3-beta-glucosidase 7
5	AD_ch12_At.12	*GbM_A12G3007*	hevamine-A-like
5	AD_ch19_Dt.05	*GbM_D05G0704*	aspartyl protease family protein
5	AD_ch25_Dt.06	*GbM_D06G2312*	metacaspase-9-like
5	AD_ch23_Dt.09	*GbM_D09G1021*	glucan endo-1,3-beta-glucosidase 7-like
9	AD_ch01_At.01	*GbM_A01G2154*	endochitinase 1-like
9	AD_ch01_At.01	*GbM_A01G0926*	probable serine/threonine-protein kinase *WNK11*
9	AD_ch04_At.04	*GbM_A04G0524*	basic endochitinase
9	AD_ch06_At.06	*GbM_A06G0181*	early nodulin-like protein 2
9	AD_ch07_At.07	*GbM_A07G0334*	class V chitinase-like
9	AD_ch09_At.09	*GbM_A09G0935*	glucan endo-1,3-beta-glucosidase
9	AD_ch09_At.09	*GbM_A09G1018*	Pesticidal crystal *cry1Ag*
9	AD_ch10_At.10	*GbM_A10G2108*	pathogenesis-related 5 protein
9	AD_ch11_At.11	*GbM_A11G1509*	proteinase inhibitor
9	AD_ch12_At.12	*GbM_A12G1835*	F-box/kelch-repeat protein *At1g67480*
9	AD_ch13_At.13	*GbM_A13G2387*	plant intracellular Ras-group-related LRR protein 6
9	AD_ch15_Dt.01	*GbM_D01G2083*	endochitinase 1 precursor
9	AD_ch15_Dt.01	*GbM_D01G0806*	cytochrome P450 *716B1*-like
9	AD_ch17_Dt.03	*GbM_D03G1889*	cytochrome P450 *87A3*-like
9	AD_ch22_Dt.04	*GbM_D04G0310*	kunitz trypsin inhibitor 5-like
9	AD_ch22_Dt.04	*GbM_D04G0309*	kunitz trypsin inhibitor 5
9	AD_ch19_Dt.05	*GbM_D05G2153*	protein *RADIALIS*-like 6
9	AD_ch19_Dt.05	*GbM_D05G1448*	peroxiredoxin-2B isoform X1
9	AD_ch25_Dt.06	*GbM_D06G0607*	hevamine-A-like
9	AD_ch25_Dt.06	*GbM_D06G2312*	metacaspase-9-like
9	AD_ch16_Dt.07	*GbM_D07G0647*	pathogenesis-related protein *PR-4*-like
9	AD_ch16_Dt.07	*GbM_D07G0723*	probable serine/threonine-protein kinase *yakA*
9	AD_ch16_Dt.07	*GbM_D07G0676*	cytochrome P450 *71A1*-like isoform X1
9	AD_ch24_Dt.08	*GbM_D08G2582*	F-box/kelch-repeat protein *At1g67480*
9	AD_ch23_Dt.09	*GbM_D09G1021*	glucan endo-1,3-beta-glucosidase 7-like
9	AD_ch20_Dt.10	*GbM_D10G2093*	*PR10-12*-like protein
9	AD_ch20_Dt.10	*GbM_D10G2099*	glucan endo-1,3-beta-glucosidase
9	AD_ch21_Dt.11	*GbM_D11G1517*	Inhibitor of trypsin and hageman factor
9	AD_ch18_Dt.13	*GbM_D13G0563*	proline-rich receptor-like protein kinase *PERK2*

**Table 5:** Potential plant defense related DEGs and chromosomal locations for the *G. barbadense* genotype GB 713 evaluated on the fifth and ninth day after inoculation (DAI) with reniform nematodes and analyzed using three reference genomes.

**Pima 90 Genome as Reference**			
**DAI**	**Chromosome**	**Gene Name**	**Function**
5	AD_ch05_At.05	*GbM_A05G0375*	cytochrome P450 *CYP72A616*-like
5	AD_ch06_At.06	*GbM_A06G0499*	F-box/kelch-repeat protein
5	AD_ch12_At.12	*GbM_A12G3007*	hevamine-A-like
5	AD_ch25_Dt.06	*GbM_D06G0476*	F-box/kelch-repeat protein
9	AD_ch01_At.01	*GbM_A01G1118*	*MLO* protein homolog 1-like
9	AD_ch05_At.05	*GbM_A05G2226*	cytochrome P450 *82C4*-like
9	AD_ch09_At.09	*GbM_A09G1049*	glucan endo-1,3-beta-glucosidase 7
9	AD_ch10_At.10	*GbM_A10G0696*	peroxidase 7
9	AD_ch11_At.11	*GbM_A11G2800*	peroxidase 44-like
9	AD_ch11_At.11	*GbM_A11G3728*	receptor-like protein *EIX2*
9	AD_ch23_Dt.09	*GbM_D09G1021*	glucan endo-1,3-beta-glucosidase 7-like
9	AD_ch21_Dt.11	*GbM_D11G2826*	peroxidase 44-like
**BARBREN 713 Genome as Reference**			
**DAI**	**Chromosome**	**Gene Name**	**Function**
5	AD_ch05_At.05	*At_05-gene-3.477*	cytochrome P450 *CYP72A616*-like
5	AD_ch12_At.12	*At_12-gene-96.39*	cytochrome P450 *81Q32*-like
5	AD_ch16_Dt.07	*Dt_07-gene-5.54*	serine/threonine-protein kinase *OXI1*
9	AD_ch01_At.01	*At_01-gene-100.114*	*MLO* protein homolog 1-like
9	AD_ch09_At.09	*At_09-gene-64.336*	glucan endo-1,3-beta-glucosidase 7
9	AD_ch10_At.10	*At_10-gene-7.50*	peroxidase 7
9	AD_ch15_Dt.01	*Dt_01-gene-5.178*	receptor-like protein 6 isoform *X1*
9	AD_ch19_Dt.05	*Dt_05-gene-63.305*	Meloidogyne-induced cotton 3
9	AD_ch23_Dt.09	*Dt_09-gene-19.151*	glucan endo-1,3-beta-glucosidase 7-like
9	AD_ch21_Dt.11	*Dt_11-gene-51.219*	peroxidase 44-like
**BARBREN 713-32 as Reference**			
**DAI**	**Chromosome**	**Gene Name**	**Function**
9	AD_ch01_At.01	*At_01-gene-20.498*	*MLO* protein homolog 1-like
9	AD_ch09_At.09	*At_09-gene-24.22*	glucan endo-1,3-beta-glucosidase 7
9	AD_ch10_At.10	*At_10-gene-7.264*	peroxidase 7

**Table 6:** Comparison of DEGs and chromosomal locations for *Gossypium arboreum* genotypes A2-100 and A2-190 on the fifth (D5) and ninth (D9) day after inoculation with reniform nematodes using ShiXiYa 1 as the reference genome.

A2-100		A2-190		Gene	Chromosome	Start	End	Function
D5	D9	D5	D9					
X		X		*XM_017782684*	A02	1298795	1300159	UPF0496 protein *At2g18630*-like
X		X		*XM_017767345*	A02	105682841	105683108	branched-chain-amino-acid aminotransferase 7
X		X		*XM_017747283*	A05	29599195	29599804	peroxidase P7-like isoform X1
X		X		*XM_017750577*	A05	30376304	30376620	Meloidogyne-induced cotton 3
X		X		*XM_017750341*	A05	89503079	89503555	Meloidogyne-induced cotton 4
X		X		*XM_053027919*	A05	89571758	89572242	uncharacterized protein *LOC108465726*
X		X		*XM_017748838*	A05	89590047	89590369	uncharacterized protein *LOC108451104*
X		X		*XM_017750393*	A05	89599822	89600285	uncharacterized protein *LOC108452593*
X		X		*XM_017748839*	A05	89612187	89612501	uncharacterized protein *LOC108451105*
X		X		*XM_053030089*	A06	5639583	5639992	metacaspase-9-like
X		X		*XM_017753691*	A10	87991737	87992813	early nodulin-75-like
X		X		*XM_017781201*	A12	104966554	104967371	phosphate transporter *PHO1* homolog 3
X		X		*XM_017781096*	A12	117838875	117840143	hevamine-A-like
X		X		*XM_017763090*	A13	114337094	114338671	epidermis-specific secreted glycoprotein *EP1*
X	X		X	*XM_017773121*	A05	4219994	4220308	aspartyl protease

X: gene differentially expressed.

**Table 7:** Comparison of DEGs and chromosomal locations for Gossypium *barbadense* genotypes TX 110 and GB 713 on the fifth (D5) and ninth (D9) day after inoculation with reniform nematodes using Pima 90 as the reference genome.

TX 110		GB 713		Gene	Chromosome	Start	End	Function
D5	D9	D5	D9					
X	X		X	*GbM_D12G2729*	AD_ch26_Dt.12	57615470	57618613	hypothetical protein *ES319_D12G259600v1*
X			X	*GbM_D12G0682*	AD_ch26_Dt.12	10834831	10836774	salicylic acid methyltransferase
	X		X	*GbM_A04G0525*	AD_ch04_At.04	11976349	11976916	Oxygen regulatory *nreC*
X		X		*GbM_A02G0072*	AD_ch02_At.02	540903	547723	Proton pump interactor 1-like protein
X		X		*GbM_A01G0832*	AD_ch01_At.01	11656447	11658118	UDP-glycosyltransferase *73C6*-like
X			X	*GbM_A09G1049*	AD_ch09_At.09	56423776	56429056	glucan endo-1,3-beta-glucosidase 7
X	X		X	*GbM_D09G1021*	AD_ch23_Dt.09	34826370	34829892	glucan endo-1,3-beta-glucosidase 7
	X		X	*GbM_A13G1710*	AD_ch13_At.13	91646679	91648528	probable glutamate dehydrogenase 3
X	X	X	X	*GbM_A11G3523*	AD_ch11_At.11	107574208	107574928	Zinc finger 77
X	X	X		*GbM_A12G3007*	AD_ch12_At.12	100301797	100303022	hevamine-A-like
X	X	X	X	*GbM_A10G2857*	AD_ch10_At.10	109107899	109109793	hypothetical protein *ES319_A10G249100v1*
X		X		*GbM_D01G0797*	AD_ch15_Dt.01	10301341	10303017	UDP-glycosyltransferase *73C6*

X: gene differentially expressed.

## Data Availability

Original RNAseq data are available upon request.
